# Structure of an Ultrathin Oxide on Pt_3_Sn(111) Solved by Machine Learning Enhanced Global Optimization**

**DOI:** 10.1002/anie.202204244

**Published:** 2022-04-29

**Authors:** Lindsay R. Merte, Malthe Kjær Bisbo, Igor Sokolović, Martin Setvín, Benjamin Hagman, Mikhail Shipilin, Michael Schmid, Ulrike Diebold, Edvin Lundgren, Bjørk Hammer

**Affiliations:** ^1^ Materials Science and Applied Mathematics Malmö University 20506 Malmö Sweden; ^2^ Center for Interstellar Catalysis Department of Physics and Astronomy Aarhus University 8000 Aarhus Denmark; ^3^ Institute of Applied Physics TU Wien 1040 Vienna Austria; ^4^ Department of Surface and Plasma Science Faculty of Mathematics and Physics Charles University 180 00 Prague 8 Czech Republic; ^5^ Div. of Synchrotron Radiation Research Lund University 22100 Lund Sweden

**Keywords:** Density Functional Calculations, Machine Learning, Structure Elucidation, Surface Chemistry

## Abstract

Determination of the atomic structure of solid surfaces typically depends on comparison of measured properties with simulations based on hypothesized structural models. For simple structures, the models may be guessed, but for more complex structures there is a need for reliable theory‐based search algorithms. So far, such methods have been limited by the combinatorial complexity and computational expense of sufficiently accurate energy estimation for surfaces. However, the introduction of machine learning methods has the potential to change this radically. Here, we demonstrate how an evolutionary algorithm, utilizing machine learning for accelerated energy estimation and diverse population generation, can be used to solve an unknown surface structure—the (4×4) surface oxide on Pt_3_Sn(111)—based on limited experimental input. The algorithm is efficient and robust, and should be broadly applicable in surface studies, where it can replace manual, intuition based model generation.

## Graphical abstract

Machine learning techniques can be implemented to accelerate surface structure determination based on density functional theory. The application of such an algorithm is demonstrated here for a surface oxide on Pt_3_Sn(111) which had eluded determination by experimental methods.

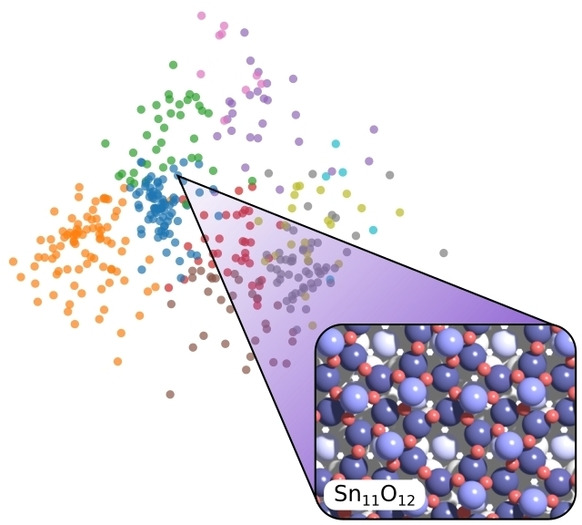

## Introduction

The atomic structure of surfaces and interfaces critically underpins our understanding of the performance of various materials, from heterogeneous catalysts and electrocatalysts to semiconductor electronics and photovoltaics. Despite the enormous importance of such fundamental information and the great advances in experimental instrumentation over the past decades, structure determination for complex surface reconstructions and ultrathin films remains a significant challenge, and many structures remain unsolved. Surface crystallography is labor intensive and prone to error, even when relatively simple structures are considered; complexity, disorder and heterogeneity can easily render such problems intractable. This naturally hinders our ability to interpret experiments that aim to establish structure‐performance relationships for these materials, and especially our ability to predict and explain such relationships through quantum chemical modelling.

The oxidation of platinum‐tin alloys provides a clear example of these limitations. Though the materials are of importance for a variety of catalytic processes,[^1^, ^2^, ^3^, ^4^, ^5^, ^6^] the structures of oxides formed on their surfaces and the details of metal‐oxide interfaces involved remain poorly understood at the atomic level. This is despite several attempts, using a variety of techniques, to characterize experimentally the well‐defined oxide layers formed on single crystal surfaces.[^7^, ^8^, ^9^, ^10^, ^11^, ^12^] A promising solution is to couple experiments closely with atomic structure prediction based on theory‐driven global optimization. Density functional theory (DFT) based global optimization strategies have played an increasing role in structure prediction efforts for more than a decade, partly replacing labor intensive, intuition guided strategies. For a review see Ref. [13]. The global optimization approaches employed encompass a diverse set of methods such as simulated annealing,^[14]^ random structure search,^[15]^ basin hopping,^[16]^ minima hopping,^[17]^ metadynamics,^[18]^ parallel tempering Monte Carlo,^[19]^ particle‐swarm optimization,^[20]^ and evolutionary strategies.[^21^, ^22^, ^23^] These optimization methods have gradually improved to handle increasingly complex problems for systems ranging from crystals to surfaces and nanoparticles etc.[^13^, ^24^]

Applicability of the global optimization methods at the density functional theory level to larger systems is, however, limited by the computational cost encountered. This is most pronounced for systems involving a large number of atoms, as is the case for surfaces, which require inclusion of a certain number of bulk layers.[^25^, ^26^, ^27^] As a solution, a new generation of structure prediction methods[^28^, ^29^, ^30^, ^31^] have achieved orders of magnitude reductions in computation time by partly replacing the expensive DFT potential with a machine‐learned approximation refined during the search.

We demonstrate here the successful application of such a machine learning enhanced algorithm to the problem of an oxidized platinum‐tin surface. Specifically, we employ the recently developed GOFEE algorithm^[31]^ which can be summarized as:

- Evaluate a few random structures at the DFT level.
- Construct a surrogate energy landscape based on all structures evaluated at the DFT level. The landscape is described with a Gaussian Process Regression (GPR) model based on a global descriptor of the structures.
- Collect a sample of the most stable, yet significantly different, structures calculated at the DFT level, see Figure 1
  **Figure 1:**
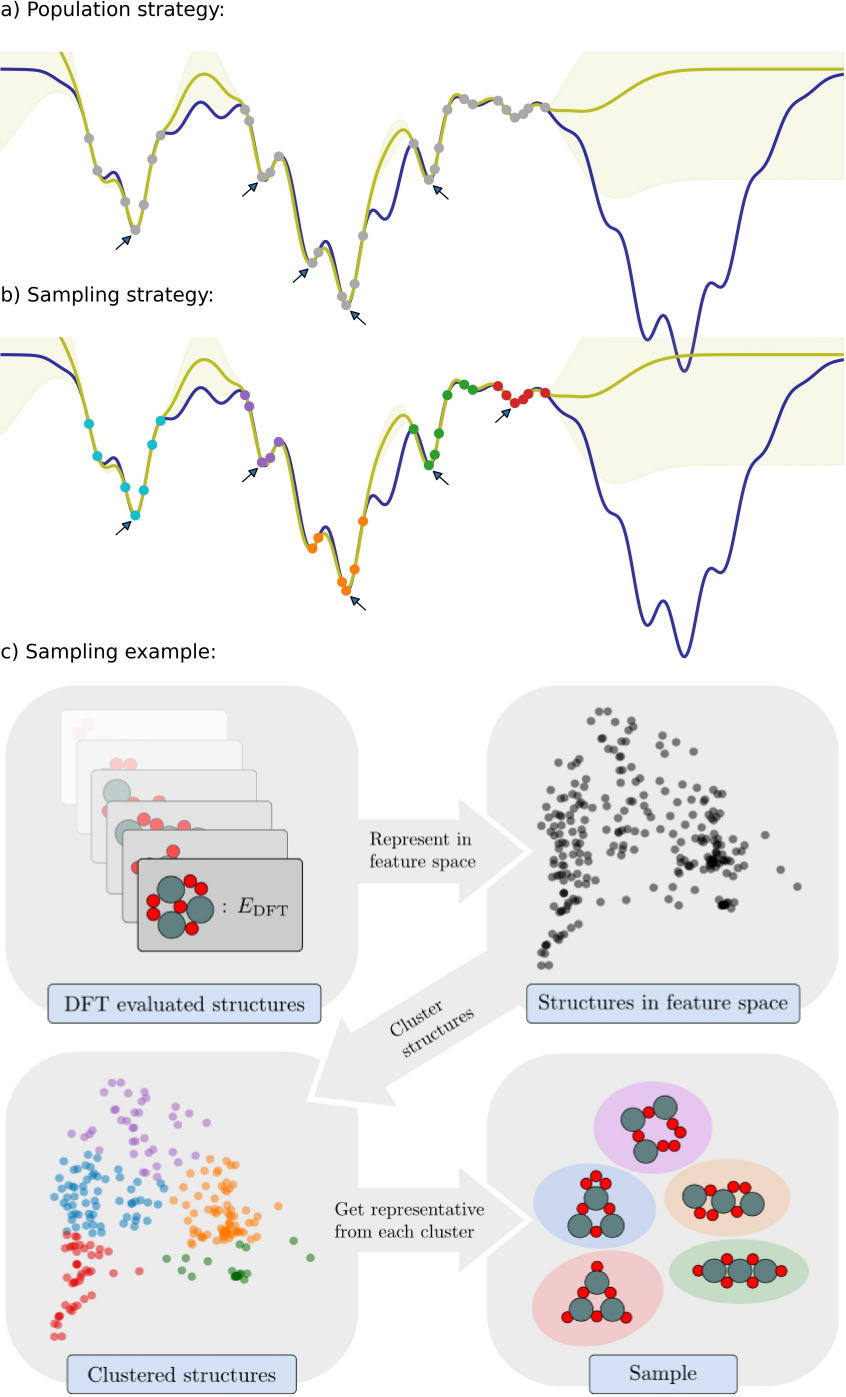
a), b) A one‐dimensional energy landscape (blue) is sampled at some select points (grey points) and a GPR model (yellow) is established. a) With an evolving population, locally optimal data points (arrows) being sufficiently different will constitute the population and not necessarily represent all data. b) With a clustering‐based sample scheme enforcing locally optimal data points (arrows) to be drawn from different clusters (colored points) a more representative sample of data is obtained. Using such a sample thus has potential to evolve more exploratively compared to a population based search. In this example, descendants from the data drawn from the red cluster are expected to more easily evolve into the right part of the energy landscape. c) Illustration of the sample generation scheme based on data from a GOFEE search for 2D Sn_3_O_6_ nano‐clusters with *N*_sample_=5. First, the current set of DFT evaluated structures are represented in a feature space and are subsequently clustered using the k‐means++ algorithm, to identify groups of related structures. The sample is then created by selecting the most stable structure from each group.
  .
- Generate multiple new structural candidates via rattle mutations applied to the sample members.
- Relax the new candidates in the lower confidence bound landscape given by *E*_LCB_=*E*−*κσ*, where *E* and *σ* are the GPR model energy and uncertainty, respectively, and where *κ* is a constant.
- Select the relaxed candidate with the lowest *E*_LCB_ and perform evaluation at the DFT level (no relaxation).
- Jump to (ii).

Details of the descriptor, the model, the sampling method, and the setup for the DFT calculations are given in the methods section of the Supporting Information.

The GOFEE method accomplishes a massive increase in efficiency while maintaining accuracy at the ab initio level mainly through two measures: Firstly, all local relaxations are done in an inexpensive machine learned landscape rather than in the expensive DFT landscape. Secondly, by selecting the most promising candidate based on comparing the relaxed lower confidence bounds, the *E*_LCB_’s, of all relaxed candidates, the method is driven by the uncertainty estimates from the surrogate model. It thereby effectively performs a Bayesian selection with (minus) *E*_LCB_ being the acquisition function and with configurational space being represented discretely by the relaxed candidates.^[31]^

In its original formulation, the GOFEE method has an evolving *population* of structures, which is implemented by deciding at every iteration if a new candidate, just evaluated at the DFT level, should replace one member of the population. In the present work, the population is replaced with a *sample* drawn from the set of all structures evaluated at the DFT level. The sample indeed plays the role of a population, but differs in that it assesses *all* DFT data in every iteration and in having potential to change faster than an evolving population. The conceptual difference between a population and a sample‐based approach is sketched in Figure 1a, b.

The scheme for obtaining the sample is illustrated in Figure 1 c, based on an actual search for the optimal structure of a 2D Sn_3_O_6_ cluster. The sample is generated from the full set of structures evaluated by DFT so far, after exclusion of those more than a fixed energy Δ*E* above the current lowest‐energy structure. The remaining structures are represented in feature space using the same global descriptor as in the GPR model. They are subsequently divided using the k‐means++ clustering algorithm,^[32]^ which arranges them into families sharing similar characteristics. The sample is finally generated by selecting the lowest‐energy member of each family, promoting convergence to multiple local structural minima. A comparison of the performance difference between a population‐based and a k‐means sample‐based GOFEE approach is given in the Supporting Information, Figure S1.

Aside from one previous study,^[33]^ where clustering was employed to modify the fitness function used for deciding which population members to extract for offspring creation, we are not aware of other work applying clustering to improve population diversity in evolutionary algorithm based atomistic structure search. Clustering has successfully been used to promote diversity in other fields.[^34^, ^35^, ^36^] In these works, however, clustering was applied only to the very recent search history and much of the data accumulated during the search was thus left unused. The current approach, in contrast, retains the entire search history and thus utilizes all available data. This ensures structural diversity during the search, further improving reliability, and enables identification of the targeted surface structure among global optimum structures found for a range of compositions. A number of applications of clustering for visualization of data for materials and molecules have also been suggested. For a review of the topic, see Ref. [37].

## Results and Discussion

**Figure 2:**
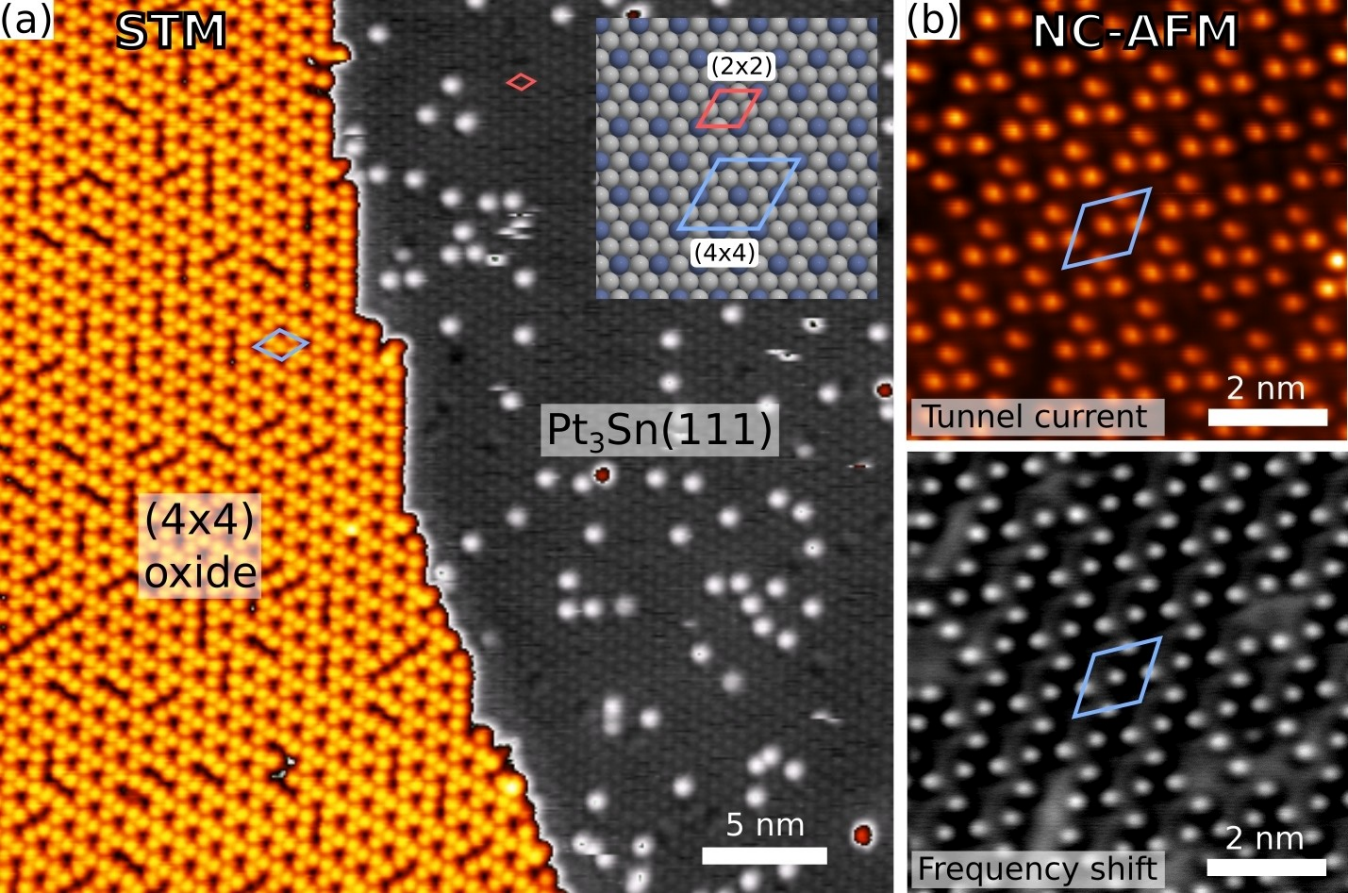
a) Scanning tunneling micrograph showing the (4×4) oxide phase partially covering the Pt_3_Sn(111) surface. Inset is a ball model of the metal surface showing the unit cell dimensions of the alloy surface and the surface oxide. b) Conductive non‐contact atomic force micrographs of the (4×4) phase showing the tunnel current and frequency shift acquired simultaneously.

The system under consideration here is the (111) surface of Pt_3_Sn, the structure of which is shown in Figure 2a. This surface has been identified as having particular relevance for electrocatalytic reactions,^[38]^ and serves as a useful model for realistic materials. Oxidation of this surface results in formation of a well ordered oxide with (4×4) periodicity relative to the underlying face centered cubic metal lattice. Scanning probe microscope (SPM) images of the oxide (Figure 2) show an array of protrusions, 3 per unit cell, in a chiral arrangement suggesting the symmetry of plane group *p*3.

Attempts at structure determination for this phase have been made using several techniques, including scanning tunneling microscopy (STM), low energy electron diffraction (LEED), X‐ray photoelectron spectroscopy and diffraction (XPS/XPD) and low energy ion scattering (LEIS),[^10^, ^11^] and further attempts have been made to characterize very similar tin oxide phases formed by deposition and oxidation of Sn on Pt(111).[^7^, ^9^] Though it has been established that the structure is terminated by tin and oxygen,^[11]^ the tin in the structure shows an XPS binding energy very close to that of tin in the alloy, hindering characterization of tin in the oxide and leading to the suggestion that most of the tin in the structure is in fact still alloyed with platinum, in a so‐called “quasimetallic” state.^[9]^ The dominance of only three protruding atoms—of unknown type—per unit cell in scanning probe micrographs and the absence of other features that would guide the construction of atomic models has further hindered structure determination by direct deduction.

For the GOFEE based structure search, we assumed a (4×4) periodic unit cell and a substrate consisting of bulklike Pt_3_Sn(111). The approximate numbers of Sn and O atoms to be placed on this substrate were chosen by assuming that the (4×4) phase consists of a Sn^2+^ oxide monolayer of some sort. Here inspiration was taken from bulk SnO and SnS, which are composed of van der Waals sheets, with Sn^2+^ in 4‐fold and 3‐fold coordination, respectively. Supported on Pt_3_Sn(111), such sheets would exhibit tin densities between 8 and 13 atoms per (4×4) unit cell. With this density range in mind and supposing somewhat higher coverages of oxygen compared to tin due to presumed oxygen affinity of tin in the substrate, we selected 16 combinations, with 9–12 Sn and 11–14 O per cell. The Sn : O composition of the (4×4) phase is hence not deduced from experiment, but rather the structure search algorithm is being applied to a range of Sn : O combinations, and afterward subjected to thermodynamic analysis and comparison with experimental data to identify a likely candidate.

**Figure 3:**
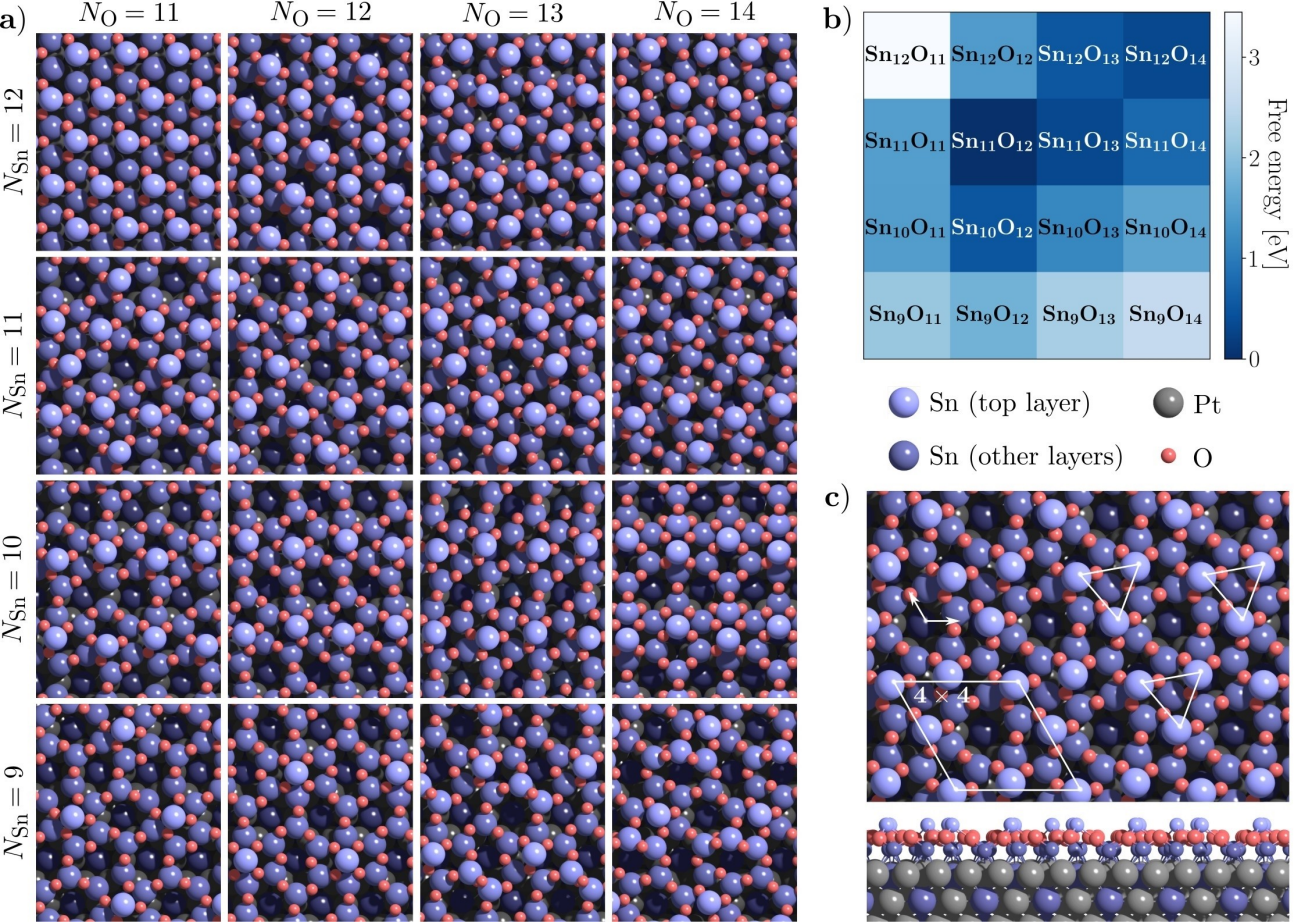
a) Minimum energy structures found by the search algorithm for various compositions of Sn and O on Pt_3_Sn(111). b) Calculated free energies for the different structures under the experimental conditions (10^−5^ mbar, 600 °C), relative to the Sn_11_O_12_ structure. c) Model of the lowest‐energy Sn_11_O_12_ structure, corresponding to the observed (4×4) phase.

Figure 3 summarizes the results of the GOFEE search performed for each of the 16 Sn : O compositions. The most stable structures found are depicted in Figure 3a and estimates of the free energies of the different structures under the conditions of the experiment are shown in Figure 3b. Importantly, a distinct minimum of these free energies is present for the Sn_11_O_12_ structure represented by the darkest blue color in Figure 3b.

For each composition considered the search was repeated four times from independent starting configurations to check for consistency. For all but three compositions (Sn_9_O_11_, Sn_9_O_13_ and Sn_9_O_14_), the same minimum‐energy structures were found in all four runs of the search. Most of the structures found exhibit a common feature: a minority of Sn atoms protrude from the surface and show a lateral spacing of ≈5–6 Å, consistent with SPM images of the (4×4) and related phases. The remaining Sn atoms are at the metal interface, with O atoms forming a layer in between. One of the 16 structures found exhibits the *p*3 symmetry expected from experiment: that with a composition Sn_11_O_12_, with three protruding Sn atoms, 8 Sn atoms at the interface, and 12 nearly coplanar O atoms in between. This structure is the very same that was found to be the most stable according to the free energy diagram, Figure 3b. The structure is further illustrated in Figure 3c.

**Figure 4:**
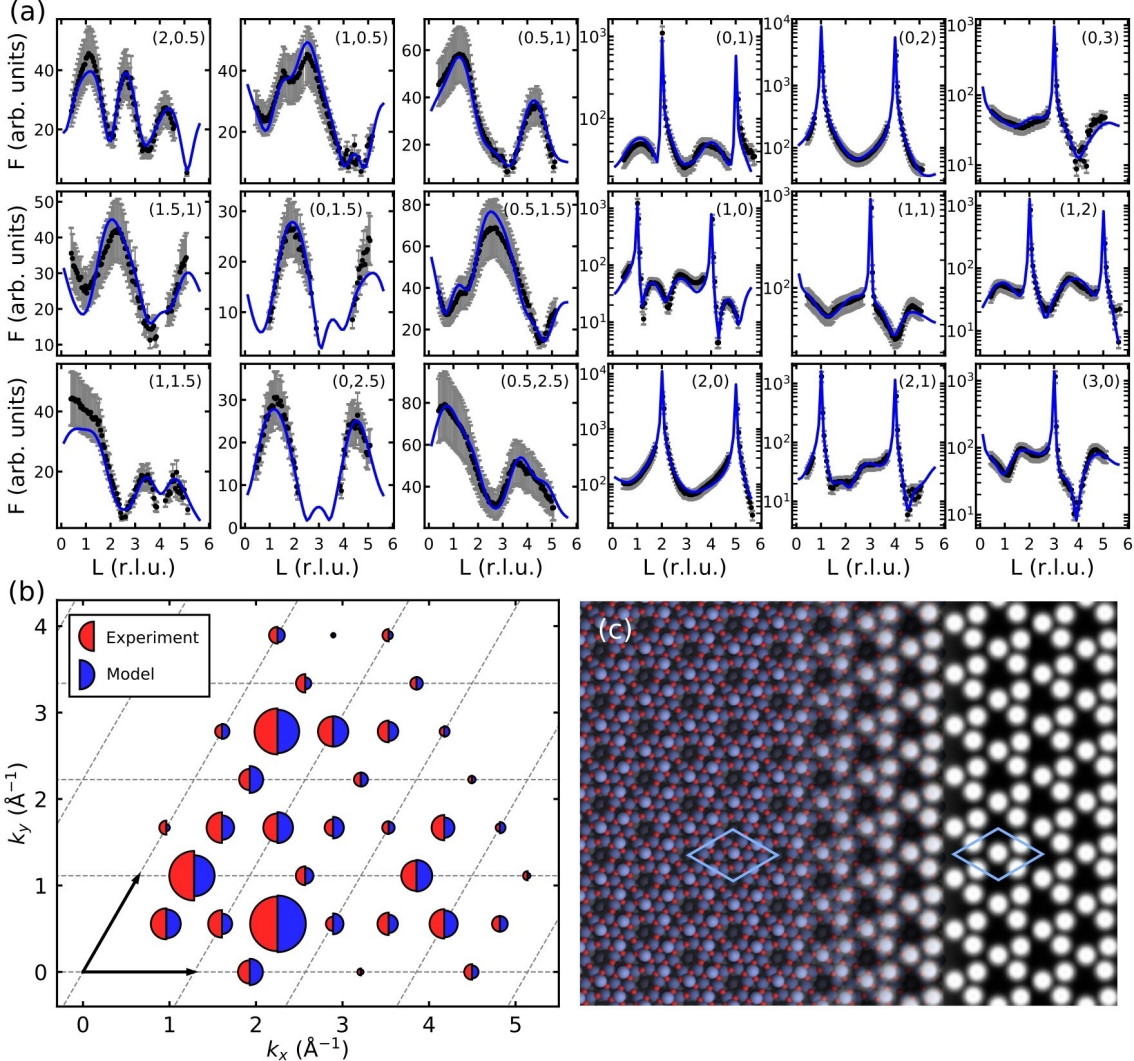
a) Measured (black) and fitted (blue) X‐ray diffraction rod profiles for the (4×4) phase. b) Reciprocal space map showing the measured and calculated in‐plane X‐ray structure factors for the (4×4) phase. Dashed lines and arrows indicate the (2×2) unit cell of the ordered alloy surface used as a reference. c) Simulated AFM frequency‐shift image of the Sn_11_O_12_ structure, based on DFT calculations.^[39]^

Surface X‐ray diffraction (SXRD) confirms this structure and enables further refinement. Figure 4a shows fits to measured rod profiles following structure refinement with the theory‐based Sn_11_O_12_ structure as the starting point. The fits to the experimental rod profiles are excellent, and the in‐plane structure factors calculated for the relaxed model reproduce the experimental ones as well (Figure 4b). During refinement, the overlayer atoms moved toward the surface by 0.1–0.2 Å, consistent with the well‐known underbinding of the employed DFT functional, but the final structure is otherwise nearly identical to that produced by the search algorithm. Simulated AFM images for the structure (Figure 4c) also show good agreement with experiment, exhibiting the characteristic chiral pattern with three protrusions per unit cell, corresponding to the three protruding Sn atoms in the structure.

The (4×4) phase can be described as a Sn^2+^ surface oxide—a contiguous two‐dimensional network related to SnO, but with a structure strongly influenced by bonding with the metal substrate. The basic structural element is Sn in 3‐fold oxygen coordination, with a strongly asymmetrical pyramidal geometry. This geometry differs somewhat from that found in bulk SnO, a layered material composed of buckled SnO sheets where Sn adopts a 4‐fold pyramidal geometry. The trigonal pyramidal geometry found here is nevertheless typical of Sn^2+^, as seen in halides and in tin(II) sulfide, and attributed to the occupation of a stereoactive lone pair orbital derived from the Sn 5 s state. This geometry has also been observed or predicted in oxidic phases, including layered, mixed‐valence Sn_3_O_4_,^[40]^ in polyoxometalate clusters,^[41]^ and in the surface layers of reduced SnO_2_(101)^[42]^ and SnO_2_(110).^[26]^

Bonding between Sn in the oxide layer and Pt in the substrate is also typical of Sn^2+^. The strength of these bonds is reflected in outward buckling of surface Pt (opposite to the clean Pt_3_Sn(111) surface, where outward buckling of Sn is observed^[43]^), and in contracted Pt−Sn bond lengths similar to what is found in organometallic clusters.[^44^, ^45^] The solution of the structure of the well‐ordered (4×4) phase thus yields the likely generalization that strong interfacial Pt‐Sn^2+^ bonding is a characteristic feature of Pt/SnO interfaces. The particular structure found here results from optimization of these bonds at the (111) surface, constrained by preferred Sn−O bond geometry.

Although these structural features can be rationalized rather easily in hindsight, the structure itself could not be directly deduced from experiment or guessed based on chemical intuition, primarily due to its multi‐layered arrangement and relatively low symmetry. An automated search method based on theory can, however, be used to generate physically plausible structural candidates for comparison with experiment, leading to the correct structure model. This methodology depends on a search algorithm that is both efficient and reliable. Our strategy utilizes the full history of the evolutionary search both to accelerate structure evaluation and to maintain a sufficiently diverse population so that premature convergence is avoided. With this we take a step towards optimal search algorithms in which all components leverage the available data to maximum benefit.

The (4×4) surface oxide on Pt_3_Sn(111) is relatively complex, but it nevertheless represents a rather ideal case, where quantitative diffraction measurements can be used to verify the result of the structure search. In general, surfaces can exhibit considerable degrees of disorder, with various coexisting phases and minority structures that cannot be characterized in detail by averaging techniques. The combination of scanning probe microscopy with theoretical simulations is often the only viable methodology in these cases, resulting in considerable uncertainty and occasionally gross misinterpretation. A reliable global optimization method like GOFEE can enable surface studies of this type to be conducted with much greater confidence.

Broad application of this approach, however, will also require the ability to characterize larger‐scale features like defects and superstructures. For this, the search algorithms must be able to scale toward hundreds of atoms, resulting in drastically more demanding searches due to the combinatorial scaling of the configuration space and the increased cost of DFT evaluations. This will require even more effective data utilization in search strategies, where the efforts presented in this work, along with recent advances in transferable machine learning potentials[^46^, ^47^, ^48^] and reinforcement learning protocols^[49]^ may pave the way for such scalable approaches. Avenues for continued progress in search methodologies are thus foreseen to ensure improved thoroughness and efficiency of automated structural search methods.

## Conclusion

Surface structure determination is a notoriously difficult problem due to the limitations of experimental techniques and the high cost of accurate theoretical modeling. In this work we have demonstrated how a machine learning driven search algorithm can be used to overcome such limitations, through the characterization of an experimentally observed (4×4) surface oxide on Pt_3_Sn(111). For this application, we further introduced in the evolutionary search method a shift from an evolving population to a k‐means based sample. The latter leverages the full history of structures visited in the search and appears highly robust and efficient for the present system.

## Supporting Information

Methods, Figures S1 to S4, Table S1.

## Conflict of interest

Authors declare that they have no competing interests.

1

## Supporting information

As a service to our authors and readers, this journal provides supporting information supplied by the authors. Such materials are peer reviewed and may be re‐organized for online delivery, but are not copy‐edited or typeset. Technical support issues arising from supporting information (other than missing files) should be addressed to the authors.

Supporting Information

## Data Availability

The data that support the findings of this study are available from the corresponding author upon reasonable request.
