# Supplementary material for: Structure of an Ultrathin Oxide on Pt3Sn(111) Solved by Machine Learning Enhanced Global Optimization
Source: Angew Chem Int Ed Engl. 2022 Apr 29;61(25):e202204244. doi: 10.1002/anie.202204244 (PMC9320988; doi:10.1002/anie.202204244)
Supplement: Supplementary file 1 — Supporting Information [file ANIE-61-0-s001.pdf]

Angewandte Chemie

Eine Zeitschrift der Gesellschaft Deutscher Chemiker

Supporting Information

Structure of an Ultrathin Oxide on $Pt_3Sn(111)$ Solved by Machine Learning Enhanced Global Optimization

L. R. Merte, M. K. Bisbo, I. Soković, M. Setvín, B. Hagman, M. Shipilin, M. Schmid, U. Diebold, E. Lundgren, B. Hammer**

Author Contributions

Conceptualization: LRM, MKB, UD, EL, BjH; Investigation: LRM, MKB, IS, MSe, BeH, MSh, MSch, EL; Methodology: MKB, BjH; Writing—original draft: LRM, MKB, IS, BjH; Writing—review and editing: LRM, MKB, IS, MSe, MSch, UD, EL, BjH.

Supplementary Information

Content included:

Methods
Figs. S1 to S4
Table S1

Methods

GOFEE details

For the evolutionary structure search, we employed the GOFEE method which detailed in Ref.[31] The machine learned energy landscape was constructed based on the global fingerprint feature from Oganov and Valle.[50] A Gaussian process regression model was built utilizing the same kernel as in Ref.[31] The kernel has two squared exponential terms with different characteristic length scales, whose values together with that of the maximal covariance were found via optimization of the marginal likelihood.

In the present work, a sample of prior DFT structures replaces the population used in the original work.[31] The sample is constructed with the k-means++ clustering method using the euclidian distance between the global fingerprint features as the distance measure between structures. All DFT structures with energies within ΔE_{sample} of the most stable structure found so far are used in the clustering. We use $\Delta E_{sample} = 5$ eV as structures of larger energy are not expected to represent interesting regions of configuration space. The sample size, N_{sample}, was chosen to 10 for the surface oxide structures as this proved efficient. Previous use of the GOFEE method has been successful with a similar size for the population. For illustrative purposes, $N_{sample} = 5$ is used in Fig. 1. Introducing the present k-means based sampling method as opposed to the original evolving population method eliminates the parameter, k_{max}, that decides how different a new population member should be from existing population members to be adopted. Figure S1 displays a comparison of the two methods showing that the k-means based method is both faster and more reliable than the population based method (for two different choices of k_{max}). Figure S2 illustrates the actual composition of a sample from one of the conducted GOFEE searches for $Sn_{11}O_{12}$.

In each episode, 200 new independent candidates were constructed via rattling of the atoms in the sampled structures. From these, the best candidate according to $E_{\rm LCB} = E - \kappa\sigma$, where

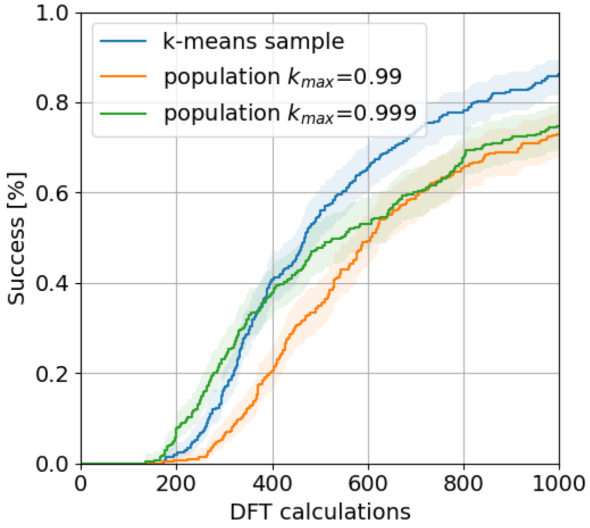

Figure S1: **Comparison of k-means based sampling with an evolving population.** The search for the $Sn_{11}O_{12}$ structure was restarted 300 times using either the new k-means based sampling or the original evolving population. For the latter, two different criteria for how similar a structure may be to other population members were used, either having max 0.99 or max 0.999 kernel elements between any two structures. Success is considered achieved when a structure has a total energy within 0.2 eV of that of the global minimum energy structure. The shaded regions represent 95% confidence intervals. The new sample-based method is both faster and more reliable. For instance, $\sim$67% of the independent restarts find the GM in less than $\sim$600 episodes, while for the evolving population based approach, $\sim$800 episodes are needed for the same fidelity. Similarly, the new method achieves 86% success after 1000 episodes, while the old method only achieves just short of 75% success. The structure searches were conducted for GOFEE implemented in a modified code base. They used 24 candidates and one DFT calculation per episode. The DFT calculation were sped up by having 1 k-point and 300 eV energy cutoff for the plane waves.

E and σ are the model energy and uncertainty, respectively, was chosen. For the constant, κ we used the value 2.

In the DFT evaluation step of GOFEE, we employed the original double-step procedure, where two single-point DFT calculations are performed. First, one is done for the candidate structure as just emerging from the acquisition. Next, another single-point DFT calculation is done for the structure modified by $\vec{F}\,\Delta x$, where $\vec{F}$ is the DFT force just calculated for the first structure, and where Δx is a step length. This proceedure seeks to provide data for the machine

learned landscape that encodes the proper direction of the energy gradient, and hence enables efficient relaxation in this surrogate energy landscape.

Population

In the main text Fig. 1, two dimensional tin oxide nano-clusters were used as an accessible example to present the new population scheme. To support this discussion we here present the application of the method to the oxidized $Pt_3Sn(111)$ surface. This is depicted in Fig. S2, where the clustering and subsequent population extraction have specifically been applied to the 500 first structures of a GOFEE search for the $Sn_{11}O_{12}$ surface composition. The figure shows the data is clustered into families of related structures along with example structures for some of these families. The families include variations in the number and arrangement of protruding Sn atoms, variations in the placement of the oxide layer with respect to the underlying metal surface, and alternative arrangements of Sn such as those in a square configuration with alternating up and down Sn, resembling strips from bulk SnO.

DFT calculations

GOFEE searches were carried out on a fixed support, consisting of two layers of $Pt_3Sn(111)$. DFT evaluations during the searches were performed using the Atomic Simulation Environment (ASE)[51] with the grid-based projector-augmented wave (GPAW) code[52,53] in plane wave mode with an energy cutoff of 400 eV and a $(2 \times 2 \times 1)$ k-point grid. Generalized gradient approximation (GGA) with the PBE functional[54] was used to describe the exchange–correlation interaction. The four best candidates for each composition was subsequently transferred to a five layer support and relaxed, fixing only the bottom two layers, using a $(4 \times 4 \times 1)$ k-point grid and a 500 eV energy cutoff until all atomic forces were below 0.05 eV/Å. Fig. 3 reports the best candidate for each composition.

Four independent GOFEE searches were carried out for each of the 16 compositions, to al-

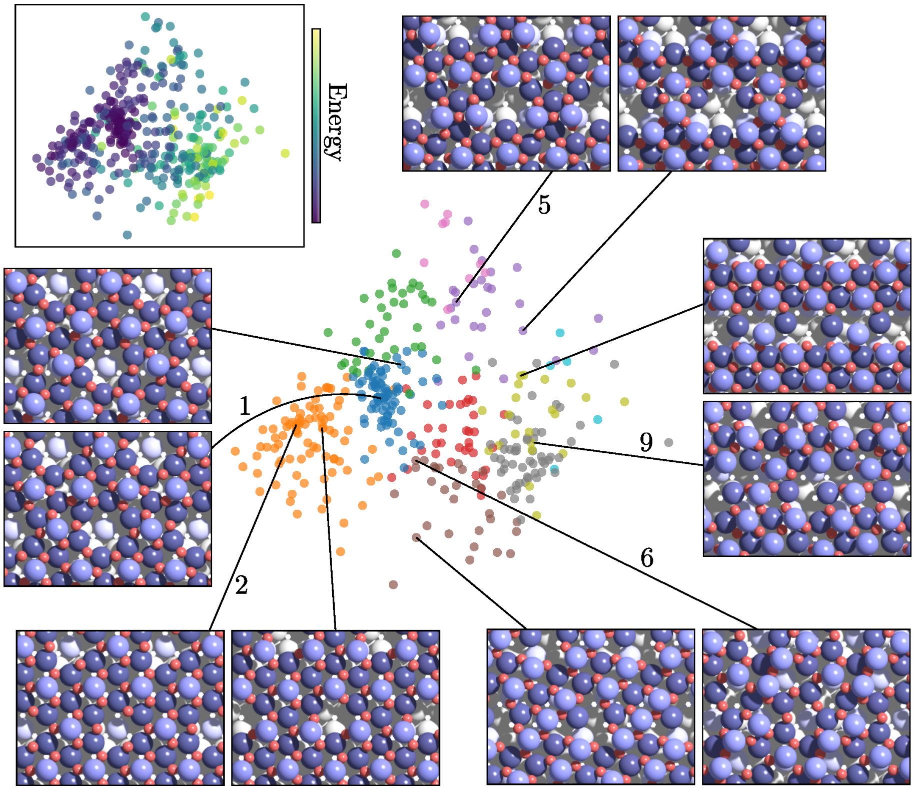

Figure S2: **Sketch of the adopted population scheme** applied to data from the first 250 iterations (500 structures) of a GOFEE search on the $Sn_{11}O_{12}$ surface composition. The scheme considers all structures evaluated so far, with an energy within $\Delta E_{sample} = 5\ eV$ of the currently lowest energy structure found. The upper left plot depicts a feature space representation of these structures, projected onto two dimensions using principal component analysis (PCA), and colored according to energy. In the center, the same structures are colored according to a clustering performed in the full feature space using the k-means algorithm. Example structures are shown for some of the clusters with atoms in the slab dimmed to highlight structural differences. The population is formed by selecting the lowest-energy structure from each cluster. The five enumerated example structures are part of the population for this particular data set and clustering. The enumeration is according to energy, with structure 1 being lowest in energy. This is coincidentally also the global minimum structure for this composition. The PCA dimensionality reduction captures 76% of the variance in the data, with the remainder (the other dimensions) accounting for the apparent overlap of clusters in the figure.

low the consistency of the searches to be evaluated. For most compositions, the assumed global minimum structure was identified in all four searches. As exceptions, it was only reproduced thrice for Sn_9O_{13}, twice for Sn_9O_{11} and once for Sn_9O_{14}.

Stability comparison

To compare the thermodynamic stability across the different compositions, and following Reuter and Scheffler,[55] the surface γ free energy was calculated for each structure, as

$$\gamma = \frac{1}{A}\left[E_{slab}(T,p) - N_{Sn}\mu_{Sn}(T,p) - N_O\mu_O(T,p) - PV - TS\right] \tag{1}$$

where A is the surface area in the computational cell, E_{slab} is the total energy of the relaxed surface slab, N_{Sn} and N_O denote, respectively, the number of tin and oxygen atoms in the slab, and finally μ_{Sn} and μ_O denote the corresponding chemical potentials. Contributions from the pressure and entropy terms are neglected.[55] Similarly, temperature and pressure contributions are neglected when evaluating the chemical potential of μ_{Sn}, such that $\mu_{Sn}(T,p) = \mu_{Sn}$. With this, the surface free energy can be simplified to

$$\gamma = \frac{1}{A}\left[E_{slab}(T,p) - N_{Sn}\mu_{Sn} - N_O\mu_O(T,p)\right] \tag{2}$$

To estimate μ_{Sn}, we assume the surface to be in equilibrium with the Pt_3Sn bulk phase, which supplies the tin atoms and turns into bulk Pt_7Sn. This gives

$$\mu_{Sn} = (7 * E_{Pt_3Sn} - 3 * E_{Pt_7Sn})/4 \tag{3}$$

Finally, assuming the O_2 atmosphere to form an ideal gas reservoir, the chemical potential of oxygen is taken from[55] to be

$$\mu_O = \frac{1}{2}E_{O_2} + \Delta\mu_O(T,p^0) + \frac{1}{2}k_BT\ln(\frac{p}{p^0}) \tag{4}$$

where E_{O_2} is the energy of the isolated molecule and $\Delta\mu_O(T,p^0)$ is the size of the temperature and pressure dependent contribution to the chemical potential at temperature T and pressure p^0, which is tabulated in.[55]

The surface free energy, γ, is related to the free energy per (4×4) cell, as given in Fig. 3, by

$$\gamma = E_{4\times 4}/A_{4\times 4}. \tag{5}$$

Surface X-ray diffraction

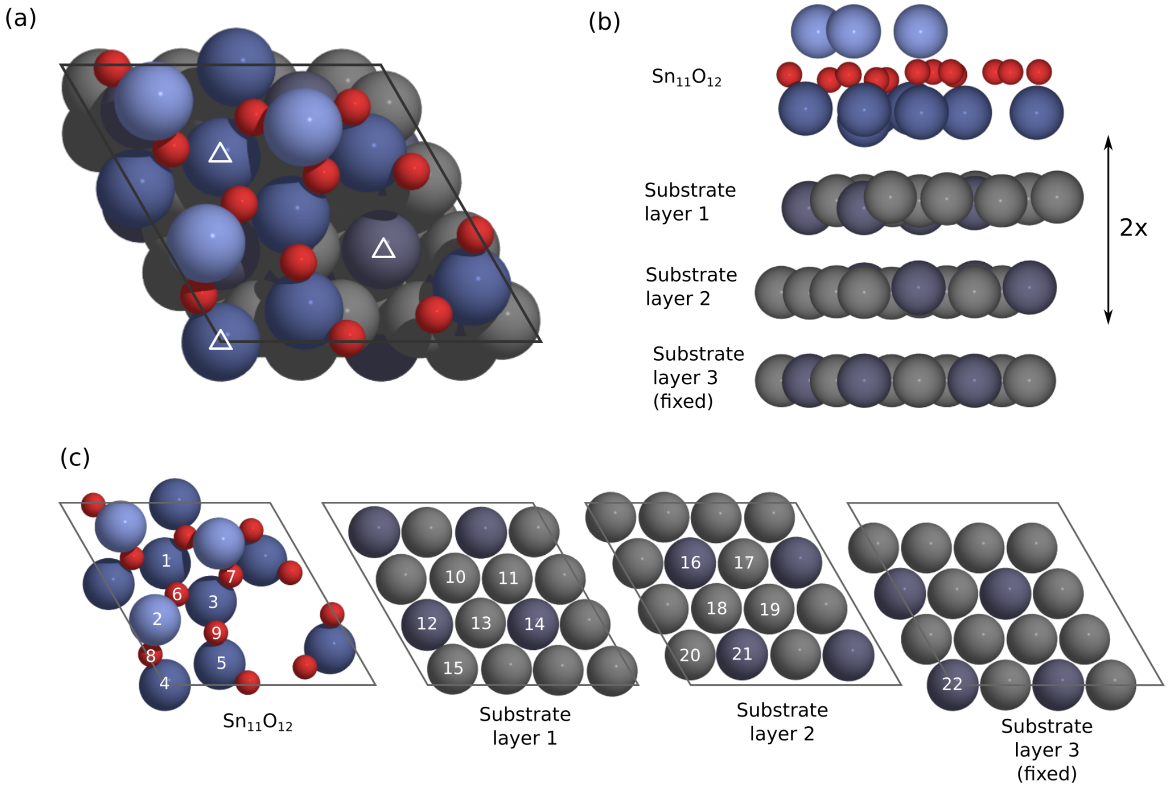

Figure S3: **Structure of the** $(4{\times}4)$ **$Sn_{11}O_{12}$ surface oxide** (a) Top view of the $(4{\times}4)$ phase, showing the unit cell and the locations of 3-fold symmetry axes. (b) Side view of the $(4{\times}4)$ phase, shown with expanded z-coordinates to highlight corrugation within the layers. c) Individual layers in the structure. Labels indicate symmetrically distinct atoms with coordinates given in Table S1.

SXRD measurements were performed at the I07 beamline at Diamond Light Source, where the sample was prepared and characterized in an ultra-high vacuum system equipped with facilities for ion sputtering, annealing and low-pressure gas exposure.[56] The sample was a 6 mm diameter $Pt_3Sn(111)$ single crystal (Mateck, GmbH) prepared by cycles of Ar^+ sputtering (1 keV) and annealing (∼800°C, 5 min) followed by oxygen exposure (10^{-5} mbar, ∼600°C, 15 min) to form the (4×4) surface oxide. $Pt_3Sn(111)$ prepared under these conditions consists of a combination of bulklike grains as well as tin-depleted inclusions consisting essentially of Pt(111)

terminated by a characteristic $(\sqrt{3}\times\sqrt{3}R30^{\circ})$ surface alloy.[11] These phases, as well as superstructures formed on them, can be distinguished in SXRD due to the difference in lattice parameter and only diffraction signals from the $Pt_3Sn(111)$ surfaces were used for the analysis here. SXRD measurements were acquired at room temperature. Crystal truncation rods and superstructure rods were measured with stationary L-scans using a Pilatus 100K area detector. Structure factors were extracted by integration of the 2D peak in each image corresponding to the intersection of the diffraction rod with the Ewald sphere, using a 'seed-skew' peak search algorithm as described by Drnec et al.[57] Structure factors were derived from raw intensities after application of the appropriate polarization and Lorentz correction factors.[57,58] In-plane structure factors were extracted from rocking scans taken at $L = 0.5$.

Fitting of SXRD data was performed using a kinematic computation of the surface structure factors[59] and least-squares minimization as implemented in SciPy.[60] Atomic coordinates were constrained to *p3* symmetry during optimization, with the initial positions derived from the DFT-optimized coordinates after symmetric averaging. The formation of *p3* overlayers on the *p3m1* $Pt_3Sn(111)$ surface implies the presence of two mirrored domains producing overlapping diffraction rods, and the fitted curves are computed as incoherent superpositions of the structure factors for the two domains. Individual intensity factors were included in the fitting of each rod and a single Debye-Waller parameter was assumed for all atoms. Due to their relatively small contribution to the overall X-ray scattering cross sections, the oxygen coordinates were constrained to those of the DFT optimization, except for an allowed vertical relaxation of the O layer as a whole. For the purpose of fitting, constant uncertainties in the measured values of 20% were assumed. The resulting rod fit yielded a reduced $\chi^2 = 0.7$. In-plane structure factors were fitted afterward, using the final, fixed coordinates from the rod fitting and allowed variation of only an overall intensity factor and an in-plane Debye-Waller parameter. The correspondence between experimental and simulated patterns for the structure is good, with $R = 0.15$. The best-fit structure is depicted in Figure S3, with coordinates presented in Table S1. Fits to SXRD

#	Element	x (Å)	y (Å)	z (Å)	x (fract.)	y (fract.)	z (fract.)
1	Sn	*0.00*	*6.52*	6.69	*0.333*	*0.667*	0.967
2	Sn	-0.53	3.48	8.96	0.131	0.356	1.296
3	Sn	2.46	4.47	6.98	0.447	0.457	1.010
4	Sn	*0.00*	*0.00*	6.95	*0.000*	*0.000*	1.005
5	Sn	2.93	1.13	6.87	0.318	0.116	0.994
6	O	*0.64*	*4.85*	*7.71*	*0.304*	*0.496*	*1.115*
7	O	*3.53*	*5.87*	*7.92*	*0.613*	*0.600*	*1.145*
8	O	*-0.78*	*1.64*	*7.85*	*0.014*	*0.168*	*1.135*
9	O	*2.73*	*2.80*	*7.92*	*0.385*	*0.287*	*1.145*
10	Pt	1.46	5.73	4.62	0.422	0.586	0.668
11	Pt	4.27	5.69	4.66	0.669	0.582	0.674
12	Sn	-0.02	3.22	4.43	0.163	0.329	0.641
13	Pt	2.84	3.32	4.61	0.421	0.340	0.667
14	Sn	*5.65*	*3.26*	4.56	*0.667*	*0.333*	0.659
15	Pt	1.34	0.85	4.71	0.162	0.086	0.681
16	Sn	*0.00*	*6.52*	2.34	*0.333*	*0.667*	0.339
17	Pt	2.84	6.50	2.28	0.584	0.664	0.330
18	Pt	1.41	4.10	2.25	0.334	0.419	0.325
19	Pt	4.23	4.07	2.28	0.583	0.417	0.330
20	Pt	0.00	1.61	2.27	0.082	0.165	0.328
21	Sn	2.78	1.64	2.39	0.330	0.168	0.346
22	Sn	*0.00*	*0.00*	*0.00*	*0.000*	*0.000*	*0.000*

Table S1: **Atomic coordinates of the $Sn_{11}O_{12}$ structure**. Fractional coordinates given with respect to the hexagonal unit cell indicated in Fig. S3, with $a = b = 11.29$ Å, $c = 6.92$ Å, $\alpha = \beta = 90°, \gamma = 120°$. Grayed values indicate parameters that were fixed during fitting. In the case of the oxygen atoms, lateral positions were fixed to those of the DFT structure, while a single displacement parameter was allowed to shift the positions in the vertical direction.

data were also attempted for a platinum-skin model, where Sn in the topmost metallic layer is substituted with Pt. The fit result was notably worse, with χ^2 increasing by 70%, to 1.1, and $R = 0.26$ for the in-plane structure factors. Although experiments indicate that a similar $(4{\times}4)$ phase is formed on pure platinum surfaces[9] which is probably the same $Sn_{11}O_{12}$ phase found here, under our experimental conditions the oxide forms atop bulk-terminated $Pt_3Sn(111)$.

STM and AFM measurements

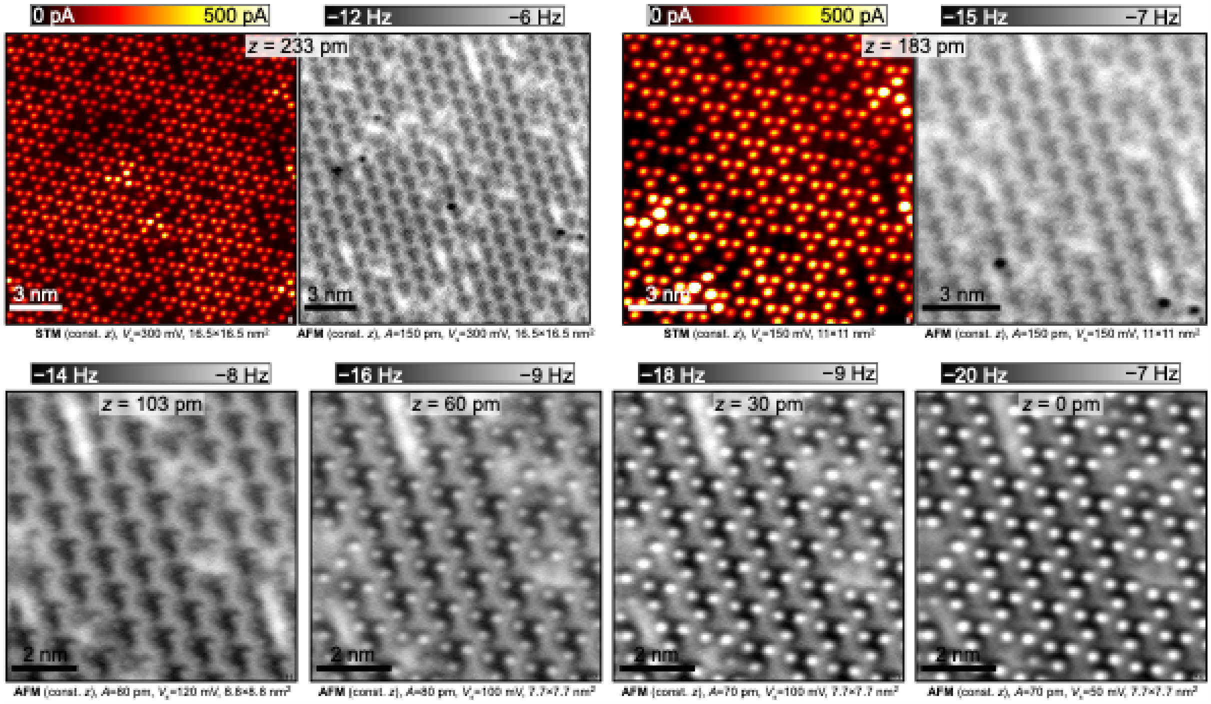

Figure S4: **AFM/STM measurements with varying tip heights.** Sequence of AFM images acquired in constant-height mode as the tip was stepped successively closer to the sample surface. Tip heights are given relative to that acquired at the smallest distance. Short-range interactions are exclusively repulsive between the tip and the protruding Sn, as indicated by the increasingly positive frequency shift (bright contrast).

Scanning tunneling microscopy (STM) characterization was performed using an Omicron VT STM located at the MAX IV Laboratory, Lund, Sweden. Measurements were acquried at room temperature with an etched W tip in constant current mode. The sample was prepared in

the same manner as in the SXRD experiments.

Atomic force microscopy (AFM) was performed at the Vienna University of Technology (TU Wien) using an Omicron LT-STM equipped with a qPlus sensor, a W tip, and custom preamplifier.[61] Measurements were acquired at $\sim$5 K after a similar sample preparation procedure. At short tip-sample distances (bottom images in Fig. S4), frequency shift images showed an array of protrusions matching what is imaged by STM, and the interactions of the tip with these atoms was exclusively repulsive.

For the AFM simulations, the $Sn_{11}O_{12}$ structure was modeled with DFT calculations using the Vienna Ab initio Simulation Package (VASP)[62,63] using the generalized gradient approximation (GGA) within the Perdew, Burke, and Ernzerhof parametrization.[54] The slab contained 48 Pt atoms, 27 Sn atoms, and 12 O atoms, placed in a $11.29\times11.29\times35.0$ Å^3 cell, with $11.29\times11.29\times23.5$ Å^3 volume of vacuum. The reciprocal space was mapped with a $4\times4\times1$ mesh of k-points. The $6s^15d^9$ Pt, $5s^25p^2$ Sn, and $2s^22p^4$ O valence electrons were explicitly modeled, while the remaining (core) electrons were modeled using the corresponding, element-specific pseudo-potentials. The criterium for ionic convergence was set to 0.01 eV/Å, together with the strong criterium for electronic convergence. The electrostatic potential was generated such that it includes the ionic potential, the Hartree contribution and the exchange-correlation potential.

Constant-height AFM images were simulated by calculating the force acting on the virtual AFM tip over a range of distances from the surface. The force maps were subsequently transformed into frequency shifts according to the small oscillation amplitude approximation.[64] Three different simulations methods were applied: (i) Probing the DFT-optimized electrostatic potential above the surface with a unit point charge; (ii) the Probe Particle Model with an empirical Lennard-Jones potential modeling the interaction between the surface atoms and a negatively charged, oxygen-terminated tip;[65] and (iii) explicitly calculated DFT force-distance curves between a CO molecule above the top-protruding Sn atom of the surface, according to a

procedure implemented in ref.[39] (Here an increased vacuum volume was used.) In each case the resulting simulated AFM image corresponds well to the measured AFM images. The simulated image shown in Fig. 4c in the main text was created with method (iii) 3.9 Å away from the protruding Sn atoms.

References

[1] R. Burch, *J. Catal.* **1981**, *71*, 348 – 359.

[2] R. D. Cortright, J. M. Hill, J. A. Dumesic, *Catal. Today* **2000**, *55*, 213–223.

[3] F. Coloma, A. Sepúlveda-Escribano, J. Fierro, F. Rodríguez-Reinoso, *Appl. Catal. A* **1996**, *136*, 231–248.

[4] A. Moscu, L. Veyre, C. Thieuleux, F. Meunier, Y. Schuurman, *Catal. Today* **2015**, *258*, 241–246.

[5] M. Arenz, V. Stamenkovic, B. Blizanac, K. Mayrhofer, N. Markovic, P. Ross, *J. Catal.* **2005**, *232*, 402 – 410.

[6] C. Lamy, S. Rousseau, E. Belgsir, C. Coutanceau, J.-M. Léger, *Electrochim. Acta* **2004**, *49*, 3901–3908.

[7] M. Batzill, D. E. Beck, B. E. Koel, *Appl. Phys. Lett.* **2001**, *78*, 2766–2768.

[8] M. Batzill, D. E. Beck, B. E. Koel, *Phys. Rev. B* **2001**, *64*, 245402.

[9] M. Batzill, J. Kim, D. E. Beck, B. E. Koel, *Phys. Rev. B* **2004**, *69*, 165403.

[10] A. Atrei, U. Bardi, G. Rovida, M. Torrini, M. Hoheisel, S. Speller, *Surf. Sci.* **2003**, *526*, 193–200.

[11] M. Hoheisel, S. Speller, W. Heiland, A. Atrei, U. Bardi, G. Rovida, *Phys. Rev. B* **2002**, *66*, 165416.

[12] J. Zheng, M. Busch, L. Artiglia, T. Skála, J. Rossmeisl, S. Agnoli, *Adv. Mater. Interfaces* **2019**, *6*, 1801874.

[13] A. Oganov, C. Pickard, Q. Zhu, R. Needs, *Nat. Rev. Mater.* **2019**, *4*, 331–348.

[14] S. Kirkpatrick, C. D. Gelatt, M. P. Vecchi, *Science* **1983**, *220*, 671.

[15] C. J. Pickard, R. J. Needs, *J. Phys.: Condens. Matter* **2011**, *23*, 053201.

[16] D. J. Wales, J. P. K. Doye, *J. Phys. Chem. A* **1997**, *101*, 5111–5116.

[17] M. Amsler, S. Goedecker, *J. Chem. Phys.* **2010**, *133*, 224104.

[18] A. Laio, M. Parrinello, *Proc. Natl. Acad. Sci. U.S.A.* **2002**, *99*, 12562–12566.

[19] D. J. Earl, M. W. Deem, *Phys. Chem. Chem. Phys.* **2005**, *7*, 3910–3916.

[20] Y. Wang, J. Lv, L. Zhu, Y. Ma, *Phys. Rev. B* **2010**, *82*, 094116.

[21] A. R. Oganov, C. W. Glass, *J. Chem. Phys.* **2006**, *124*, 244704.

[22] N. L. Abraham, M. I. J. Probert, *Phys. Rev. B* **2006**, *73*, 224104.

[23] L. B. Vilhelmsen, B. Hammer, *J. Chem. Phys.* **2014**, *141*, 044711.

[24] S. Woodley, G. Day, R. Catlow, *Phil. Trans. R. Soc. A* **2020**, *378*, 20190600.

[25] M. Sierka, T. K. Todorova, J. Sauer, S. Kaya, D. Stacchiola, J. Weissenrieder, S. Shaikhutdinov, H.-J. Freund, *J. Chem. Phys.* **2007**, *126*, 234710.

[26] L. R. Merte, M. S. Jørgensen, K. Pussi, J. Gustafson, M. Shipilin, A. Schaefer, C. Zhang, J. Rawle, C. Nicklin, G. Thornton, R. Lindsay, B. Hammer, E. Lundgren, *Phys. Rev. Lett.* **2017**, *119*, 096102.

[27] H. Zakaryan, A. Kvashnin, A. Oganov, *Sci. Rep.* **2017**, *7*, 10357.

[28] E. L. Kolsbjerg, A. A. Peterson, B. Hammer, *Phys. Rev. B* **2018**, *97*, 195424.

[29] V. Deringer, D. Proserpio, G. Csanyi, C. Pickard, *Faraday Discuss.* **2018**, *211*, 45–59.

[30] Q. Tong, L. Xue, J. Lv, Y. Wang, Y. Ma, *Faraday Discuss.* **2018**, *211*, 31–43.

[31] M. K. Bisbo, B. Hammer, *Phys. Rev. Lett.* **2020**, *124*, 086102.

[32] D. Arthur, S. Vassilvitskii in *Proceedings of the Eighteenth Annual ACM-SIAM Symposium on Discrete Algorithms*, Society for Industrial and Applied Mathematics, USA, of *SODA '07*, p. 1027–1035.

[33] M. S. Jørgensen, M. N. Groves, B. Hammer, *J. Chem. Theory Comput.* **2017**, *13*, 1486–1493.

[34] Y. Wang, C. Dang, H. Li, L. Han, J. Wei in *2009 IEEE Congress on Evolutionary Computation*, pp. 2927–2933.

[35] Y. Yuan, H. Xu, B. Wang, X. Yao, *IEEE Trans. Evol- Comput.* **2016**, *20*, 16–37.

[36] X. Zhang, Y. Tian, R. Cheng, Y. Jin, *IEEE Trans. Evol. Comput.* **2018**, *22*, 97–112.

[37] B. Cheng, R.-R. Griffiths, S. Wengert, C. Kunkel, T. Stenczel, B. Zhu, V. L. Deringer, N. Bernstein, J. T. Margraf, K. Reuter, G. Csanyi, *Acc. Chem. Res.* **2020**, *53*, 1981–1991.

[38] V. R. Stamenković, M. Arenz, C. A. Lucas, M. E. Gallagher, P. N. Ross, N. M. Marković, *J. Am. Chem. Soc.* **2003**, *125*, 2736–2745.

[39] I. Sokolović, M. Reticcioli, M. Čalkovský, M. Wagner, M. Schmid, C. Franchini, U. Diebold, M. Setvín, *Proc. Natl. Acad. Sci. U.S.A.* **2020**, *117*, 14827–14837.

[40] A. Seko, A. Togo, F. Oba, I. Tanaka, *Phys. Rev. Lett.* **2008**, *100*, 045702.

[41] K. Suzuki, T. Hanaya, R. Sato, T. Minato, K. Yamaguchi, N. Mizuno, *Chem. Commun.* **2016**, *52*, 10688–10691.

[42] M. Batzill, A. M. Chaka, U. Diebold, *Europhys. Lett.* **2004**, *65*, 61–67.

[43] A. Atrei, U. Bardi, G. Rovida, M. Torrini, E. Zanazzi, P. N. Ross, *Phys. Rev. B* **1992**, *46*, 1649–1654.

[44] M. Bortoluzzi, A. Ceriotti, I. Ciabatti, R. Della Pergola, C. Femoni, M. Carmela Iapalucci, A. Storione, S. Zacchini, *Dalton Trans.* **2016**, *45*, 5001–5013.

[45] M. C. Jennings, G. Schoettel, S. Roy, R. J. Puddephatt, *Organometallics* **1991**, *10*, 580–586.

[46] V. L. Deringer, N. Bernstein, G. Csányi, C. Ben Mahmoud, M. Ceriotti, M. Wilson, D. A. Drabold, S. R. Elliott, *Nature* **2021**, *589*, 59–64.

[47] J. S. Smith, B. T. Nebgen, R. Zubatyuk, N. Lubbers, C. Devereux, K. Barros, S. Tretiak, O. Isayev, A. E. Roitberg, *Nat. Commun.* **2019**, *10*, 2903.

[48] C. van der Oord, G. Dusson, G. Csányi, C. Ortner, *Mach. Learn.: Sci. Technol.* **2020**, *1*, 015004.

[49] H. L. Mortensen, S. A. Meldgaard, M. K. Bisbo, M.-P. V. Christiansen, B. Hammer, *Phys. Rev. B* **2020**, *102*, 075427.

[50] M. Valle, A. R. Oganov, *Acta Crystallogr. A* **2010**, *66*, 507–517.

[51] A. H. Larsen, J. Jørgen Mortensen, J. Blomqvist, I. E. Castelli, R. Christensen, M. Dułak, J. Friis, M. N. Groves, B. Hammer, C. Hargus, E. D. Hermes, P. C. Jennings, P. Bjerre Jensen, J. Kermode, J. R. Kitchin, E. Leonhard Kolsbjerg, J. Kubal, K. Kaasbjerg, S. Lysgaard, J. Bergmann Maronsson, T. Maxson, T. Olsen, L. Pastewka, A. Peterson, C. Rostgaard, J. Schiøtz, O. Schütt, M. Strange, K. S. Thygesen, T. Vegge, L. Vilhelmsen, M. Walter, Z. Zeng, K. W. Jacobsen, *J. Phys.: Condens. Matter* **2017**, *29*, 273002.

[52] J. J. Mortensen, L. B. Hansen, K. W. Jacobsen, *Phys. Rev. B* **2005**, *71*, 035109.

[53] J. Enkovaara, C. Rostgaard, J. Mortensen, J. Chen, M. Dulak, L. Ferrighi, J. Gavnholt, C. Glinsvad, V. Haikola, H. A. Hansen, H. H. Kristoffersen, M. Kuisma, A. H. Larsen, L. Lehtovaara, M. Ljungberg, O. Lopez-Acevedo, P. Moses, J. Ojanen, T. Olsen, V. Petzold, N. A. Romero, J. Stausholm-Møller, M. Strange, G. Tritsaris, M. Vanin, M. Walter, B. Hammer, H. Häkkinen, G. Madsen, R. Nieminen, J. Nørskov, M. Puska, T. Rantala, J. Schiøtz, K. Thygesen, K. W. Jacobsen, *J. Phys.: Condens. Matter* **2010**, *22 25*, 253202.

[54] J. P. Perdew, K. Burke, M. Ernzerhof, *Phys. Rev. Lett.* **1996**, *77*, 3865.

[55] K. Reuter, M. Scheffler, *Phys. Rev. B* **2001**, *65*, 035406.

[56] C. Nicklin, T. Arnold, J. Rawle, A. Warne, *J. Synchrotron Rad.* **2016**, *23*, 1245–1253.

[57] J. Drnec, T. Zhou, S. Pintea, W. Onderwaater, E. Vlieg, G. Renaud, R. Felici, *J. Appl. Crystallogr.* **2014**, *47*, 365–377.

[58] E. Vlieg, *J. Appl. Crystallogr.* **1997**, *30*, 532–543.

[59] E. Vlieg, *J. Appl. Crystallogr.* **2000**, *33*, 401–405.

[60] P. Virtanen, R. Gommers, T. E. Oliphant, M. Haberland, T. Reddy, D. Cournapeau, E. Burovski, P. Peterson, W. Weckesser, J. Bright, S. J. van der Walt, M. Brett, J. Wilson,

K. J. Millman, N. Mayorov, A. R. J. Nelson, E. Jones, R. Kern, E. Larson, C. J. Carey, İ. Polat, Y. Feng, E. W. Moore, J. VanderPlas, D. Laxalde, J. Perktold, R. Cimrman, I. Henriksen, E. A. Quintero, C. R. Harris, A. M. Archibald, A. H. Ribeiro, F. Pedregosa, P. van Mulbregt, SciPy 1.0 Contributors, *Nat. Methods* **2020**, *17*, 261–272.

[61] F. Huber, F. J. Giessibl, *Rev. Sci. Instrum.* **2017**, *88*, 073702.

[62] G. Kresse, J. Furthmüller, *Phys. Rev. B* **1996**, *54*, 11169.

[63] G. Kresse, J. Furthmüller, *Comput. Mater. Sci.* **1996**, *6*, 15–50.

[64] F. J. Giessibl, *Phys. Rev. B* **1997**, *56*, 16010.

[65] P. Hapala, G. Kichin, C. Wagner, F. S. Tautz, R. Temirov, P. Jelinek, *Phys. Rev. B* **2014**, *90*, 085421.
